# Requirements for Graduation in Both Medicine and Dentistry in England and Scotland

**Published:** 1886-10

**Authors:** 


REQUIREMENTS FOR GRADUATION IN BOTH MEDICINE AND DENTISTRY IN ENGLAND AND SCOTLAND.—From the London *Dental Record* we learn the following, the degrees obtained being M. R. C. S. and L. D. S:

"After the preliminary examination has been passed, the student should register as both a dental and medical student; during the mechanical apprenticeship receive instruction from any registered medical practitioner, or from any pharmaceutical chemist, or at a public hospital, or infirmary, or dispensary, in chemistry, including chemical physics, practical chemistry, pharmacy and materia medica, and present himself for examination in these before entering a hospital; or, if he prefers it, he may take the two latter later in his career, viz., at the second examination. At the expiration of his first winter let him pass in elementary anatomy and physiology; at the end of his second winter let him take anatomy and physiology.

At the expiration of two years he may present himself for the dental license; he will during these two yeais have been attending simultaneously both the general and dental hospital. During the remainder of his time he should devote himself to surgery, medicine, and midwifery, &c., in which subjects he may be examined at the expiration of two years from the time of passing the second examination.

It was felt that the recent changes brought about by the amalgamation of the two Colleges have greatly increased the difficulty of obtaining these higher qualifications; I must ask you to take my word for it that such is not really the case; the curriculum is really simplified, and a candidate now is only re-examined in the subject in which he fails. The old M. R. C. S. only, as a separate diploma, is a thing of the past; it is only advisable to deal with things as they are. I may be asked what is the extra time and money required to take these extra qualifications? It takes two more years, but the whole of these need not be necessarily spent in London. One winter and two summer sessions may be passed in one of the following ways:

(*a*) Attending the practice of a hospital, infirmary, or other institution recognized as affording satisfactory opportunities for professional study;

(*b*) Receiving instruction as a pupil of a legally qualified practitioner holding such a public appointment, or having such opportunities of imparting a practical knowledge of medicine, surgery, or midwifery, as shall be satisfactory to the two colleges;

(*c*) Attending lectures on one or more of the required subjects of professional study at a recognised place of instruction.

The twenty cases of labour can be signed for by any legally qualified practitioner.

The duties of clinical clerk and surgical dresser, which must be discharged after the second examination during six months each, can be performed at a general hospital, infirmary or dispensary, or parochial or union infirmary, recognized for this purpose.

These arrangements make it less costly for students whose parents live in large towns where such public institutions are found, a large proportion of the expenditure being living in town.

The actual increased expenditure in hospital fees is about fifty or sixty guineas. The examination fees for the three examinations for the double qualification under the conjoint scheme is thirty-five guineas.

For the convenience of reference I should like to tabulate as concisely as possible the best mode of procedure for the dental student to obtain the three examinations:—

1. Preliminary examination.
2. Apprenticeship.
3. Register as a dental and medical student, or this latter can be postponed until entry at hospital.
4. During apprenticeship receive instruction as above in chemistry, materia medica, and pharmacy, and pass in them at the College of Surgeons.
5. Enter simultaneously at a dental and general hospital.
6. Pass in elementary anatomy and physiology at end of first winter session.
7. Pass in anatomy and physiology at end of second winter session.
8. Take dental license at end of second year.
9. Devote remainder of time to medicine, surgery, midwifery, &c.
10. Pass, at expiration of two years from second examination, the final test of the two Colleges.

In conclusion, I should like to point out, side by side, the requirements of the curriculum for the dental license and that for the double qualification, thus demonstrating how much of the latter curriculum must of necessity be taken by the dental student, and how few the extra subjects required.

| *Requirements for Double Qualifications at a General Hospital.* | *Requirements for Dental license at a General Hospital.* |
|---|---|
| Anatomy—One course of lectures. | Anatomy—One course and twenty lectures on head and neck. |
| Physiology—One course of lectures, three months extra practical physiology. | Physiology—One course of lectures. |
| Dissections—Twelve months. | Dissections–Nine months. |
| Surgery—One course of lectures. | Surgery—One course of lectures. |
| Medicine—One course of lectures. | Medicine—One course of lectures. |

| | |
|---|---|
| Materia Medica, Chemistry, Practical Chemistry—Done before entering school now. | Materia Medica — One course of lectures. Chemistry — One course of lectures. Practical Chemistry--One course of lectures. |
| Practical Surgery and Medicine—Three winter and two summer sessions. | Practical Surgery and Clinical Lectures—Two winter sessions. |

*Extra Work Required.*

Midwifery—One course of lectures, and twenty labour csses.

Practical systematic instruction in medicine, surgery and mid-wifery.

Instructions and proficiency in vaccination.

Pathological Anatomy—One course of lectures and demonstrations in post-mortem room during attendance on clinical lectures.

Forensic Medicine—One course.

Clinical Lectures on Medicine—Nine months.

Ciinical Lecture on Surgery—Nine months.

Clinical Study on Midwifery—Three months.

Clinical Clerk—Six months.

Surgical Dresser—Six months."

